# Bis(2-acetylpyridine-κ^2^ *N*,*O*)silver(I) tetra­fluoridoborate: a complex with silver in a seesaw coordination geometry

**DOI:** 10.1107/S1600536810048014

**Published:** 2010-11-24

**Authors:** Michael A. O’Donnell, Peter J. Steel

**Affiliations:** a Chemistry Department, University of Canterbury, PO Box 4800, Christchurch, New Zealand

## Abstract

The reaction of 2-acetylpyridine with silver(I) tetra­fluorido­borate leads to the discrete title complex, [Ag(C_7_H_7_NO)_2_]BF_4_, in the cation of which the Ag atom is coordinated by two 2-acetylpyridine ligands, each of which is *N*,*O*-bidentate, albeit with stronger bonding to the N atoms [Ag—N = 2.2018 (15) and 2.2088 (14) Å; Ag—O = 2.5380 (13) and 2.5454 (13) Å]. The four-coordinate Ag atom has a seesaw coordination geometry with a τ_4_ index of 0.51. The tetra­fluoridoborate anion is disordered over two orientations with 0.568 (10):0.432 (10) occupancies.

## Related literature

For other silver complexes with the same ligand, see: Bowmaker *et al.* (2005 ▶); Drew *et al.* (2005 ▶); Di Nicola *et al.* (2010 ▶). For examples of our previous work on silver complexes, see: Steel (2005 ▶); Fitchett & Steel (2006 ▶); O’Keefe & Steel (2007) ▶; Steel & Fitchett (2008 ▶); Golder *et al.* (2010 ▶). For details of the coordination geometry of four-coordinate silver, see: Young & Hanton (2008 ▶). For a definition of the τ_4_ index, see: Yang *et al.* (2007 ▶). 2-acetylpyridine coordin­ates to a variety of transition metals, usually as an *N*,*O*-chelating ligand, although it has been reported to act as an *O*-monodentate donor to a zinc porphyrin, see: Byrn *et al.* (1993 ▶).
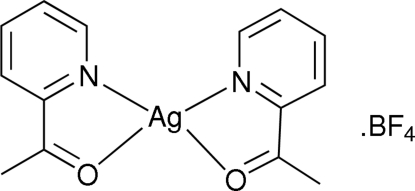


## Experimental

### 

#### Crystal data

[Ag(C_7_H_7_NO)_2_]BF_4_
*M* *_r_* = 436.95
Triclinic, 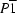

*a* = 7.2635 (2) Å
*b* = 9.7091 (3) Å
*c* = 11.7390 (4) Å
α = 85.624 (2)°
β = 81.452 (2)°
γ = 75.054 (2)°
*V* = 790.34 (4) Å^3^
*Z* = 2
Mo *K*α radiation
μ = 1.33 mm^−1^
*T* = 116 K
0.37 × 0.36 × 0.14 mm

#### Data collection

Bruker SMART CCD area-detector diffractometer
Absorption correction: multi-scan (*SADABS*; Bruker, 2009 ▶) *T*_min_ = 0.805, *T*_max_ = 1.000
18086 measured reflections
3661 independent reflections
3255 reflections with *I* > 2σ(*I*)
*R*_int_ = 0.042

#### Refinement

*R*[*F* ^2^ > 2σ(*F* ^2^)] = 0.024
*wR*(*F* ^2^) = 0.051
*S* = 1.01
3661 reflections
247 parameters
H-atom parameters constrained
Δρ_max_ = 0.33 e Å^−3^
Δρ_min_ = −0.47 e Å^−3^

### 

Data collection: *SMART* (Bruker, 2009 ▶); cell refinement: *SAINT* (Bruker, 2009 ▶); data reduction: *SAINT*; program(s) used to solve structure: *SHELXS97* (Sheldrick, 2008 ▶); program(s) used to refine structure: *SHELXL97* (Sheldrick, 2008 ▶); molecular graphics: *SHELXTL* (Sheldrick, 2008 ▶); software used to prepare material for publication: *SHELXTL*.

## Supplementary Material

Crystal structure: contains datablocks global, I. DOI: 10.1107/S1600536810048014/zs2079sup1.cif

Structure factors: contains datablocks I. DOI: 10.1107/S1600536810048014/zs2079Isup2.hkl

Additional supplementary materials:  crystallographic information; 3D view; checkCIF report

## Figures and Tables

**Table 1:** Selected bond angles (°)

N9—Ag1—N1	165.92 (6)
N9—Ag1—O15	70.09 (5)
N1—Ag1—O15	122.03 (5)
N9—Ag1—O7	121.62 (5)
N1—Ag1—O7	69.62 (5)
O15—Ag1—O7	83.23 (5)

**Fig. 1.**
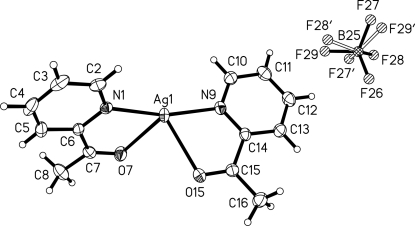
The molecular structure of the title complex, showing displacement ellipsoids at the 50% probability level.

## supplementary crystallographic information

### Comment

For some time we have been involved in the study of silver complexes of chelating and bridging heterocyclic ligands (Steel, 2005; Fitchett & Steel, 2006; O'Keefe & Steel, 2007; Steel & Fitchett, 2008; Golder *et al.*, 2010). 2-acetylpyridine coordinates to a variety of transition metals, usually as an N,*O*-chelating ligand, although it has been reported to act as an O-monodentate donor to a zinc porphyrin (Byrn *et al.*, 1993). X-ray crystal structures have been reported for complexes of 2-acetylpyridine with silver(I) perchlorate (Bowmaker *et al.*, 2005; Drew *et al.*, 2005), trifluoroacetate (Bowmaker *et al.*, 2005), trifluoromethanesulfonate (Di Nicola *et al.*, 2010) and nitrate (Bowmaker *et al.*, 2005). The latter has a single 2-acetylpyridine bound to the silver with a chelating nitrate anion, while the others have two chelating 2-acetylpyridine ligands. We now report the structure of its complex with silver(I) tetrafluoridoborate, the title compound [Ag(C_7_H_7_NO)_2_] BF_4_ (I).

In (I), the asymmetric unit contains a complex cation comprising a silver(I) atom bound to two bidentate N,*O*-chelated 2-acetylpyridine ligands [Ag—N, 2.2018 (15), 2.2088 (14) Å; Ag—O, 2.5380 (13), 2.5454 (13) Å], and a tetrafluoridoborate counter-anion (Fig. 1). The tetrafluoridoborate anion is disordered over two orientations with relative F occupancies of 57:43% about a common central B. Since the silver atom makes no other contacts less than 2.72 Å it should be classified as four-coordinate (Young & Hanton, 2008). Calculation of the τ_4_ index (Yang *et al.*, 2007) produces a value of 0.51 which means that the geometry around the silver should be described as seesaw.

### Experimental

The title compound was prepared by diffusion of pentane into a methanol solution of a mixture of 2-acetylpyridine and silver(I) tetrafluoridoborate.

### Refinement

Hydrogen atoms were included in calculated positions as riding atoms, with *U*_iso_(H) = 1.2*U*_eq_(C) for the pyridine H atoms and *U*_iso_(H) = 1.5*U*_eq_(C) for the acetyl H atoms. The occupancies for the disordered F atoms of the BF_4_ anion were 0.568 (10)/0.432 (10) and were fixed at 0.57/0.43 in the refinement.

### Crystal data

**Table:**

[Ag(C_7_H_7_NO)_2_]BF_4_	*Z* = 2
*M**_r_* = 436.95	*F*(000) = 432
Triclinic, *P*1	*D*_x_ = 1.836 Mg m^−^^3^
Hall symbol: -P 1	Mo *K*α radiation, λ = 0.71073 Å
*a* = 7.2635 (2) Å	Cell parameters from 8378 reflections
*b* = 9.7091 (3) Å	θ = 2.7–27.6°
*c* = 11.7390 (4) Å	µ = 1.33 mm^−^^1^
α = 85.624 (2)°	*T* = 116 K
β = 81.452 (2)°	Block, colourless
γ = 75.054 (2)°	0.37 × 0.36 × 0.14 mm
*V* = 790.34 (4) Å^3^	

### Data collection

**Table:**

Bruker SMART CCD area-detector diffractometer	3661 independent reflections
Radiation source: fine-focus sealed tube	3255 reflections with *I* > 2σ(*I*)
graphite	*R*_int_ = 0.042
φ and ω scans	θ_max_ = 27.6°, θ_min_ = 2.7°
Absorption correction: multi-scan (*SADABS*; Bruker, 2009)	*h* = −9→9
*T*_min_ = 0.805, *T*_max_ = 1.000	*k* = −12→12
18086 measured reflections	*l* = −15→15

### Refinement

**Table:**

Refinement on *F*^2^	Primary atom site location: structure-invariant direct methods
Least-squares matrix: full	Secondary atom site location: difference Fourier map
*R*[*F*^2^ > 2σ(*F*^2^)] = 0.024	Hydrogen site location: inferred from neighbouring sites
*wR*(*F*^2^) = 0.051	H-atom parameters constrained
*S* = 1.01	*w* = 1/[σ^2^(*F*_o_^2^) + (0.0251*P*)^2^] where *P* = (*F*_o_^2^ + 2*F*_c_^2^)/3
3661 reflections	(Δ/σ)_max_ = 0.001
247 parameters	Δρ_max_ = 0.33 e Å^−^^3^
0 restraints	Δρ_min_ = −0.47 e Å^−^^3^

### Special details

**Table:**

Geometry. All e.s.d.'s (except the e.s.d. in the dihedral angle between two l.s. planes) are estimated using the full covariance matrix. The cell e.s.d.'s are taken into account individually in the estimation of e.s.d.'s in distances, angles and torsion angles; correlations between e.s.d.'s in cell parameters are only used when they are defined by crystal symmetry. An approximate (isotropic) treatment of cell e.s.d.'s is used for estimating e.s.d.'s involving l.s. planes.
Refinement. Refinement of *F*^2^ against ALL reflections. The weighted *R*-factor *wR* and goodness of fit *S* are based on *F*^2^, conventional *R*-factors *R* are based on *F*, with *F* set to zero for negative *F*^2^. The threshold expression of *F*^2^ > σ(*F*^2^) is used only for calculating *R*-factors(gt) *etc*. and is not relevant to the choice of reflections for refinement. *R*-factors based on *F*^2^ are statistically about twice as large as those based on *F*, and *R*- factors based on ALL data will be even larger.

### Fractional atomic coordinates and isotropic or equivalent isotropic displacement parameters (Å^2^)

**Table:**

	*x*	*y*	*z*	*U*_iso_*/*U*_eq_	Occ. (<1)
Ag1	0.62372 (2)	0.358455 (15)	0.216889 (12)	0.02670 (6)	
N1	0.7431 (2)	0.40200 (16)	0.03743 (13)	0.0200 (3)	
C2	0.8515 (3)	0.2955 (2)	−0.02733 (17)	0.0266 (4)	
H2	0.8596	0.2001	0.0010	0.032*	
C3	0.9529 (3)	0.3180 (3)	−0.13419 (18)	0.0338 (5)	
H3	1.0279	0.2398	−0.1781	0.041*	
C4	0.9425 (3)	0.4558 (3)	−0.17500 (17)	0.0350 (5)	
H4	1.0125	0.4742	−0.2471	0.042*	
C5	0.8291 (3)	0.5676 (2)	−0.10994 (16)	0.0279 (5)	
H5	0.8186	0.6635	−0.1375	0.033*	
C6	0.7311 (3)	0.53789 (19)	−0.00415 (15)	0.0200 (4)	
O7	0.5413 (2)	0.62559 (14)	0.16893 (11)	0.0279 (3)	
C7	0.6095 (3)	0.65429 (19)	0.07221 (16)	0.0227 (4)	
C8	0.5744 (3)	0.8064 (2)	0.0268 (2)	0.0353 (5)	
H8A	0.4863	0.8686	0.0840	0.053*	
H8B	0.5175	0.8157	−0.0449	0.053*	
H8C	0.6966	0.8340	0.0117	0.053*	
N9	0.4948 (2)	0.26845 (16)	0.37767 (14)	0.0238 (4)	
C10	0.3564 (3)	0.1997 (2)	0.37892 (17)	0.0281 (4)	
H10	0.3117	0.1917	0.3082	0.034*	
C11	0.2753 (3)	0.1397 (2)	0.47867 (18)	0.0299 (5)	
H11	0.1757	0.0933	0.4765	0.036*	
C12	0.3420 (3)	0.1486 (2)	0.58052 (17)	0.0299 (5)	
H12	0.2899	0.1077	0.6500	0.036*	
C13	0.4870 (3)	0.2183 (2)	0.58072 (16)	0.0262 (4)	
H13	0.5360	0.2250	0.6502	0.031*	
C14	0.5589 (3)	0.27780 (19)	0.47815 (15)	0.0211 (4)	
O15	0.7480 (2)	0.43100 (16)	0.38824 (12)	0.0414 (4)	
C15	0.7082 (3)	0.3619 (2)	0.47483 (16)	0.0235 (4)	
C16	0.7981 (3)	0.3629 (2)	0.58006 (17)	0.0327 (5)	
H16A	0.6993	0.4069	0.6420	0.049*	
H16B	0.8956	0.4177	0.5638	0.049*	
H16C	0.8587	0.2647	0.6040	0.049*	
B25	0.9904 (3)	0.9500 (2)	0.7668 (2)	0.0297 (5)	
F26	1.1442 (2)	0.96995 (15)	0.81393 (12)	0.0499 (4)	
F27	1.0213 (7)	0.8050 (5)	0.7373 (6)	0.0530 (14)	0.568 (10)
F28	0.8389 (6)	0.9732 (7)	0.8535 (4)	0.0778 (17)	0.568 (10)
F29	0.9559 (8)	1.0366 (5)	0.6754 (4)	0.0486 (13)	0.568 (10)
F27'	0.8174 (6)	1.0520 (7)	0.7916 (8)	0.080 (3)	0.432 (10)
F28'	1.0393 (11)	0.9638 (11)	0.6459 (4)	0.059 (2)	0.432 (10)
F29'	0.9705 (11)	0.8213 (7)	0.7954 (6)	0.0507 (17)	0.432 (10)

### Atomic displacement parameters (Å^2^)

**Table:**

	*U*^11^	*U*^22^	*U*^33^	*U*^12^	*U*^13^	*U*^23^
Ag1	0.03524 (10)	0.02888 (9)	0.01804 (8)	−0.01335 (7)	−0.00432 (6)	0.00624 (6)
N1	0.0188 (8)	0.0235 (8)	0.0192 (8)	−0.0063 (7)	−0.0057 (6)	−0.0002 (6)
C2	0.0221 (11)	0.0319 (11)	0.0285 (10)	−0.0069 (9)	−0.0088 (8)	−0.0063 (8)
C3	0.0235 (11)	0.0533 (15)	0.0262 (11)	−0.0086 (10)	−0.0028 (8)	−0.0161 (10)
C4	0.0231 (11)	0.0685 (16)	0.0166 (10)	−0.0177 (11)	−0.0005 (8)	−0.0047 (10)
C5	0.0226 (11)	0.0441 (13)	0.0214 (10)	−0.0163 (9)	−0.0076 (8)	0.0088 (9)
C6	0.0176 (10)	0.0269 (10)	0.0183 (9)	−0.0094 (8)	−0.0073 (7)	0.0039 (7)
O7	0.0334 (8)	0.0262 (7)	0.0217 (7)	−0.0048 (6)	−0.0011 (6)	−0.0005 (6)
C7	0.0180 (10)	0.0233 (10)	0.0286 (10)	−0.0070 (8)	−0.0081 (8)	0.0038 (8)
C8	0.0332 (13)	0.0251 (11)	0.0470 (14)	−0.0087 (9)	−0.0058 (10)	0.0083 (9)
N9	0.0267 (9)	0.0215 (8)	0.0229 (8)	−0.0064 (7)	−0.0042 (7)	0.0035 (6)
C10	0.0287 (12)	0.0309 (11)	0.0270 (10)	−0.0101 (9)	−0.0074 (8)	0.0015 (8)
C11	0.0241 (11)	0.0300 (11)	0.0353 (12)	−0.0102 (9)	0.0027 (9)	−0.0001 (9)
C12	0.0320 (12)	0.0300 (11)	0.0261 (11)	−0.0109 (9)	0.0063 (8)	−0.0005 (8)
C13	0.0306 (12)	0.0272 (10)	0.0198 (10)	−0.0072 (9)	0.0008 (8)	−0.0031 (8)
C14	0.0235 (10)	0.0177 (9)	0.0198 (9)	−0.0026 (7)	−0.0008 (7)	0.0001 (7)
O15	0.0635 (11)	0.0464 (10)	0.0277 (8)	−0.0383 (9)	−0.0109 (7)	0.0100 (7)
C15	0.0271 (11)	0.0206 (9)	0.0219 (10)	−0.0050 (8)	−0.0008 (8)	−0.0030 (7)
C16	0.0323 (12)	0.0444 (13)	0.0245 (11)	−0.0153 (10)	−0.0037 (9)	−0.0002 (9)
B25	0.0255 (13)	0.0262 (12)	0.0366 (13)	−0.0086 (10)	−0.0009 (10)	0.0049 (10)
F26	0.0574 (10)	0.0577 (9)	0.0475 (9)	−0.0335 (8)	−0.0209 (7)	0.0115 (7)
F27	0.040 (2)	0.0249 (14)	0.099 (4)	−0.0053 (14)	−0.028 (2)	−0.003 (2)
F28	0.047 (2)	0.097 (4)	0.071 (3)	−0.009 (2)	0.0300 (17)	0.005 (2)
F29	0.060 (3)	0.043 (2)	0.051 (2)	−0.026 (2)	−0.022 (2)	0.0213 (17)
F27'	0.043 (3)	0.065 (4)	0.118 (7)	0.018 (2)	−0.011 (3)	−0.027 (4)
F28'	0.065 (4)	0.098 (6)	0.031 (2)	−0.053 (4)	−0.006 (2)	0.015 (3)
F29'	0.063 (4)	0.034 (3)	0.067 (4)	−0.030 (3)	−0.022 (3)	0.022 (3)

### Geometric parameters (Å, °)

**Table:**

Ag1—N1	2.2088 (14)	C10—C11	1.388 (3)
Ag1—N9	2.2018 (15)	C10—H10	0.9500
Ag1—O7	2.5454 (13)	C11—C12	1.372 (3)
Ag1—O15	2.5380 (15)	C11—H11	0.9500
N1—C2	1.338 (2)	C12—C13	1.391 (3)
N1—C6	1.357 (2)	C12—H12	0.9500
C2—C3	1.389 (3)	C13—C14	1.385 (2)
C2—H2	0.9500	C13—H13	0.9500
C3—C4	1.373 (3)	C14—C15	1.510 (3)
C3—H3	0.9500	O15—C15	1.215 (2)
C4—C5	1.385 (3)	C15—C16	1.482 (3)
C4—H4	0.9500	C16—H16A	0.9800
C5—C6	1.386 (2)	C16—H16B	0.9800
C5—H5	0.9500	C16—H16C	0.9800
C6—C7	1.505 (3)	B25—F29'	1.307 (6)
O7—C7	1.212 (2)	B25—F29	1.324 (4)
C7—C8	1.502 (2)	B25—F28	1.368 (4)
C8—H8A	0.9800	B25—F26	1.380 (3)
C8—H8B	0.9800	B25—F27'	1.393 (5)
C8—H8C	0.9800	B25—F28'	1.415 (5)
N9—C10	1.340 (2)	B25—F27	1.428 (5)
N9—C14	1.348 (2)		
			
N9—Ag1—N1	165.92 (6)	N9—C10—C11	123.05 (19)
N9—Ag1—O15	70.09 (5)	N9—C10—H10	118.5
N1—Ag1—O15	122.03 (5)	C11—C10—H10	118.5
N9—Ag1—O7	121.62 (5)	C12—C11—C10	118.66 (19)
N1—Ag1—O7	69.62 (5)	C12—C11—H11	120.7
O15—Ag1—O7	83.23 (5)	C10—C11—H11	120.7
C2—N1—C6	118.14 (16)	C11—C12—C13	119.11 (17)
C2—N1—Ag1	120.49 (12)	C11—C12—H12	120.4
C6—N1—Ag1	120.74 (13)	C13—C12—H12	120.4
N1—C2—C3	122.97 (19)	C14—C13—C12	119.07 (19)
N1—C2—H2	118.5	C14—C13—H13	120.5
C3—C2—H2	118.5	C12—C13—H13	120.5
C4—C3—C2	118.6 (2)	N9—C14—C13	122.03 (17)
C4—C3—H3	120.7	N9—C14—C15	116.76 (15)
C2—C3—H3	120.7	C13—C14—C15	121.16 (18)
C3—C4—C5	119.31 (18)	C15—O15—Ag1	110.96 (13)
C3—C4—H4	120.3	O15—C15—C16	121.06 (18)
C5—C4—H4	120.3	O15—C15—C14	120.05 (18)
C4—C5—C6	119.22 (19)	C16—C15—C14	118.84 (15)
C4—C5—H5	120.4	C15—C16—H16A	109.5
C6—C5—H5	120.4	C15—C16—H16B	109.5
N1—C6—C5	121.73 (18)	H16A—C16—H16B	109.5
N1—C6—C7	116.32 (15)	C15—C16—H16C	109.5
C5—C6—C7	121.93 (17)	H16A—C16—H16C	109.5
C7—O7—Ag1	112.42 (12)	H16B—C16—H16C	109.5
O7—C7—C8	120.55 (19)	F29—B25—F28	112.6 (3)
O7—C7—C6	120.42 (16)	F29'—B25—F26	109.0 (3)
C8—C7—C6	119.04 (17)	F29—B25—F26	111.2 (2)
C7—C8—H8A	109.5	F28—B25—F26	105.6 (3)
C7—C8—H8B	109.5	F29'—B25—F27'	111.3 (4)
H8A—C8—H8B	109.5	F26—B25—F27'	115.8 (3)
C7—C8—H8C	109.5	F29'—B25—F28'	109.9 (4)
H8A—C8—H8C	109.5	F26—B25—F28'	105.7 (3)
H8B—C8—H8C	109.5	F27'—B25—F28'	104.8 (3)
C10—N9—C14	118.07 (16)	F29—B25—F27	110.4 (3)
C10—N9—Ag1	121.89 (13)	F28—B25—F27	105.7 (3)
C14—N9—Ag1	120.00 (12)	F26—B25—F27	111.2 (3)
